# ICTV Virus Taxonomy Profile: *Coronaviridae* 2023

**DOI:** 10.1099/jgv.0.001843

**Published:** 2023-04-25

**Authors:** Patrick C. Y. Woo, Raoul J. de Groot, Bart Haagmans, Susanna K. P. Lau, Benjamin W. Neuman, Stanley Perlman, Isabel Sola, Lia van der Hoek, Antonio C. P. Wong, Shiou-Hwei Yeh

**Affiliations:** 1 PhD Program in Translational Medicine and Department of Life Sciences, National Chung Hsing University, Taichung 402, Taiwan, ROC; 2 Department of Biomolecular Health Sciences, Faculty of Veterinary Medicine, Utrecht University, Utrecht, Netherlands; 3 Department of Viroscience, Erasmus Medical Center, Rotterdam, Netherlands; 4 Department of Microbiology, School of Clinical Medicine, Li Ka Shing Faculty of Medicine, The University of Hong Kong, Hong Kong, PR China; 5 Department of Biology, Texas A&M University, College Station, Texas, 77843, USA; 6 Departments of Microbiology and Immunology, and Pediatrics, University of Iowa, Iowa City, IA 52242, USA; 7 Department of Molecular and Cell Biology, National Center for Biotechnology-Spanish, National Research Council (CNB-CSIC), Madrid, Spain; 8 Department of Medical Microbiology and Infection Prevention, Amsterdam UMC, Laboratory of Experimental Virology, Location University of Amsterdam, 1105 AZ Amsterdam, Netherlands; 9 Department of Microbiology, College of Medicine, National Taiwan University, Taipei, Taiwan, ROC

**Keywords:** *Coronaviridae*, *Letovirinae*, *Orthocoronavirinae*, *Pitovirinae*, coronavirus, ICTV Report, taxonomy, COVID-19

## Abstract

The family *Coronaviridae* includes viruses with positive-sense RNA genomes of 22–36 kb that are expressed through a nested set of 3′ co-terminal subgenomic mRNAs. Members of the subfamily *Orthocoronavirinae* are characterized by 80–160 nm diameter, enveloped virions with spike projections. The orthocoronaviruses, severe acute respiratory syndrome coronavirus and Middle East respiratory syndrome-related coronavirus are extremely pathogenic for humans and in the last two decades have been responsible for the SARS and MERS epidemics. Another orthocoronavirus, severe acute respiratory syndrome coronavirus 2, was responsible for the recent global COVID-19 pandemic. This is a summary of the International Committee on Taxonomy of Viruses (ICTV) Report on the family *Coronaviridae* which is available at www.ictv.global/report/coronaviridae.

## Virion

**Fig. 1.**
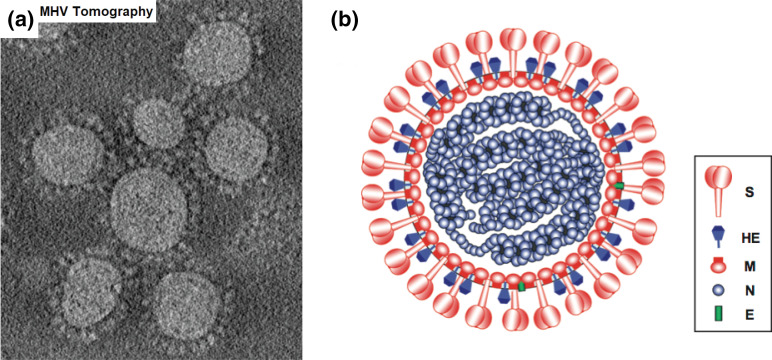
Structure of murine hepatitis virus A59 particles. (**a**) Cryoelectron tomograph of virus particles in water ice (courtesy of Ben Neuman). Virions are around 85 nm in diameter. (**b**) Schematic illustration of a particle of mouse hepatitis virus.

Orthocoronavirus virions are enveloped, pleomorphic and roughly spherical viral particles with surface projections of the spike (S) protein; the family name derives from electron micrograph images which are reminiscent of the solar corona. The nucleocapsid, comprising nucleocapsid protein (N) and RNA, appears to be loosely wound, with small helical units distributed throughout the virion interior. Virions consist of three or four membrane-associated proteins: spike (S), envelope (E) and membrane (M) glycoprotein (Table 1, Fig. 1). A haemagglutinin-esterase (HE) glycoprotein is only present in members of the subgenus *Embecovirus* in the genus *Betacoronavirus*.

**Table 1.** Characteristics of members of the family *Coronaviridae*

Example:	murine hepatitis virus A59 (AY700211), species *Murine coronavirus*, genus *Betacoronavirus*
Virion	Enveloped, pleomorphic but often quasi-spherical
Genome	22–36 kb of positive-sense, monopartite RNA
Replication	Through an antigenomic RNA generated by continuous transcription; gene expression through discontinuous transcription of a nested set of co-terminal subgenomic negative-sense RNAs which are copied into subgenomic mRNAs
Translation	From capped and polyadenylated genomic and subgenomic mRNAs
Host range	Vertebrates (mammals, birds, amphibians and fish)
Taxonomy	Realm *Riboviria*, kingdom *Orthornavirae*, phylum *Pisoniviricetes*, order *Nidovirales*, suborder *Cornidovirineae*; three subfamilies (*Letovirinae*, *Orthocoronavirinae* and *Pitovirinae*) including >5 genera and >50 species

## Genome

**Fig. 2.**
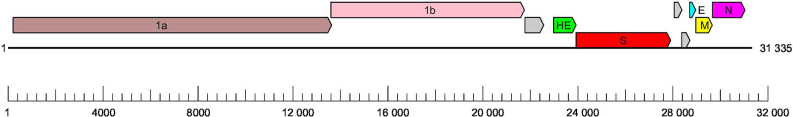
Genomic organization of murine hepatitis virus A59 (AY700211). Coloured ORFs are 1a, 1b, the proximal and distal regions of the replicase protein; S, spike protein; N, nucleocapsid; E, envelope glycoprotein; M, membrane glycoprotein; HE, haemagglutinin-esterase protein. ORFs in grey encode accessory proteins.

The coronavirus genome is a linear, positive-sense RNA of 22–36 kb with a 5′-cap structure and a 3′-polyadenylated tail [1] and a typical genome organization of 5′-NCR-replicase-S-E-M-N-NCR-3′. The replicase gene comprises two large overlapping ORFs, 1a and 1b. Translation of ORF1b is programmed by −1 ribosomal frameshifting and produces polyproteins pp1a and pp1ab, which are further processed by virus-encoded proteinases [2]. Genes for the structural proteins S, E, M and N are interspersed with a variable number of accessory protein ORFs (Fig. 2).

## Replication

Coronavirus replication takes place in the cytoplasm. The viral genome serves as a template for synthesizing genomic-size negative-sense RNA for replication and subgenomic-size negative-sense RNA molecules that template the production of subgenomic mRNAs [3]. The 3′-proximal viral genes are expressed from a nested set of 3′-coterminal subgenomic mRNAs generated by the fusion of coding sequences to common 5′-leader sequences by a mechanism of discontinuous transcription [4].

## Pathogenicity

Coronaviruses infect a wide range of animals including humans. Clinical manifestations range from asymptomatic to fatal diseases. Nine coronaviruses that infect humans have been identified so far, including four human seasonal coronaviruses (human coronavirus OC43, human coronavirus 229E, human coronavirus NL63, human coronavirus HKU1), canine coronavirus, porcine coronavirus HKU15 (also known as porcine deltacoronavirus), severe acute respiratory syndrome coronavirus, severe acute respiratory syndrome coronavirus 2 and Middle East respiratory syndrome-related coronavirus.

## Taxonomy

Current taxonomy: ictv.global/taxonomy. In the subfamily *Orthocoronavirinae*, members of the genera *Alphacoronavirus* and *Betacoronavirus* infect mammals, especially bats; members of the genera *Gammacoronavirus* and *Deltacoronavirus* primarily infect birds, but also mammals [5]. Hosts for members of the subfamilies *Letovirinae* and *Pitovirinae* are amphibians and bony fish, respectively [6].

## Resources

Full ICTV Report on the family *Coronaviridae*: ictv.global/report/coronaviridae.

## Abbreviations

E: envelope glycoprotein
HE: haemagglutinin-esterase protein
M: membrane glycoprotein
N: nucleocapsid
S: spike protein.
